# Performance and overview of clinically relevant areas of application of saliva testing in the cat

**DOI:** 10.3389/fvets.2024.1385345

**Published:** 2024-05-22

**Authors:** Maike Schroers, Andrea Meyer-Lindenberg

**Affiliations:** Clinic of Small Animal Surgery and Reproduction, Veterinary Faculty, Ludwig-Maximilians-University Munich, Munich, Germany

**Keywords:** cat, saliva, infections, allergens, physiology

## Abstract

**Introduction:**

The cat represents an important model in order to investigate basic physiological knowledge of salivary secretion as well as pharmacokinetics of active substances.

**Objective:**

The aim of the study was to review in which diagnostic application areas saliva testing is routinely used and in which areas it could be further explored in the future.

**Materials and methods:**

Literature relevant to the research question was collected in March 2022 using the Pubmed database.

**Results:**

The diagnosis of infectious diseases in cat saliva is one of the most important fields of application. Saliva diagnostics may also indicate dental diseases, allergies or kidney and other metabolic diseases. Sexual and stress hormones can also be measured in cat saliva. A number of clinically relevant allergens in cat saliva that may cause allergies in humans has been investigated and described, in addition to infectious agents that can be transmitted from cats to humans.

**Conclusions:**

Saliva testing in cats can be useful in many areas, including the detection of infectious diseases, allergies and dental disease. However, it is far from being used to its full potential within veterinary medicine.

## 1 Introduction

As the knowledge of common human and animal diseases increases, so do the possibilities for diagnosis and treatment. In the field of laboratory diagnostics, saliva diagnostics is becoming increasingly important alongside the examination of blood, cerebrospinal fluid, urine and other body fluids. Saliva is produced in the cat's oral cavity by large glands, the parotid gland (*Glandula parotis*) and the mandibular gland (*Glandula mandibularis*), as well as other small glands (1). It is 99.5% water, the other components being mucins (called glycoproteins), proteins and digestive enzymes (2). The salivary glands are very well-vascularized and innervated (3). Through the salivary ducts, saliva enters the oral cavity through their endings, called acini. The acinar cells are surrounded by an extracellular matrix, myoepithelial cells, myofibroblasts, endothelial cells, stromal cells, immune cells and nerve fibers (3). Naturally, saliva is rich in immunoglobulins (4, 5). In addition to providing digestive enzymes, saliva is primarily responsible for maintaining and preserving oral tissue.

**Figure 1:**
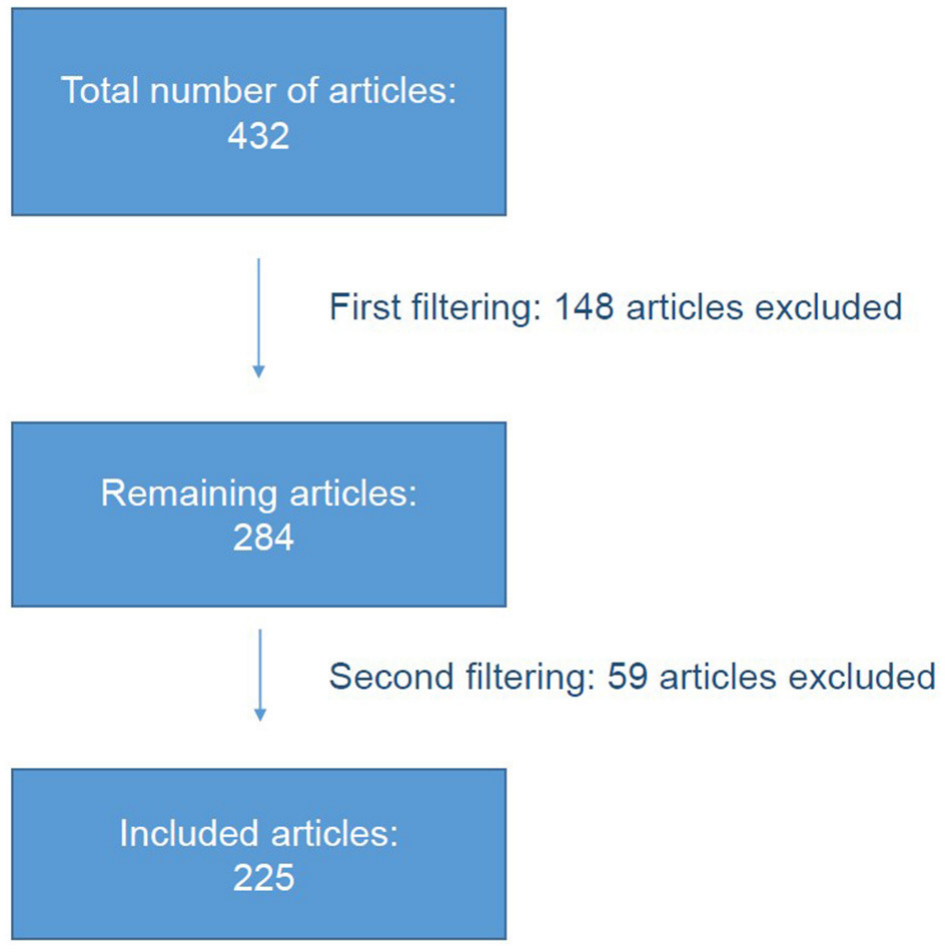
Filtering of the articles.

**Figure 2:**
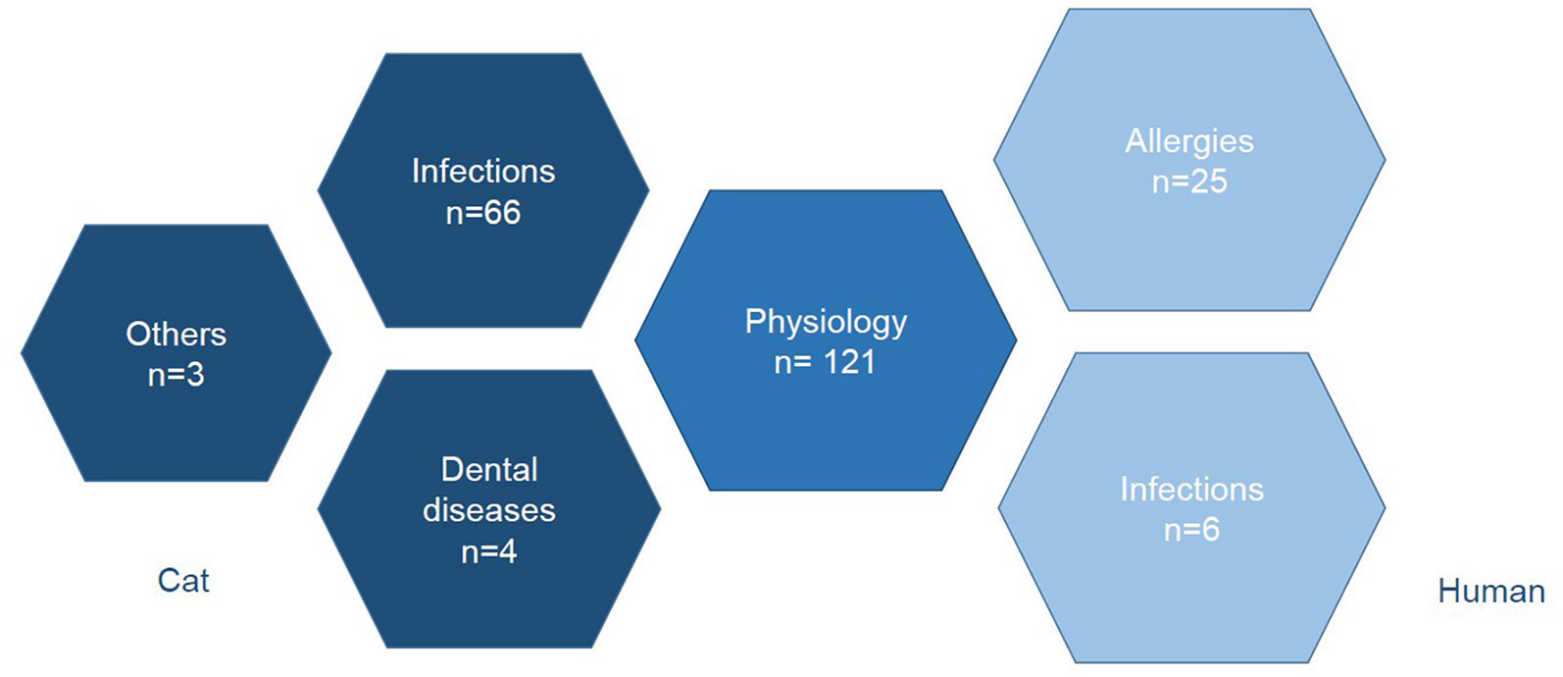
Sorting the articles into different subject areas.

This review article critically examines the areas of application of feline salivary testing in order to identify areas for further investigation in order to further improve feline health. Literature relevant to the research question was collected in March 2022 using the Pubmed database. The terms “cat” and “saliva” were searched. This was followed by the filtering process, before the articles were sorted thematically and the content was recorded. The first filtering excluded papers that did not fit the research question based on their title or content. The second filtering excluded articles that were not directly related to salivary diagnostics. Review articles were also excluded. After the filtering process, the relevant articles were sorted by topic and the content, if available, was entered into an Excel spreadsheet based on the abstract or full text. The articles were sorted by topic into different spreadsheets. A total of 432 articles were found. After filtering, 225 articles could be included in the study (Figure 1). When sorting the articles by topic, a distinction was made between studies relating to the cat and studies investigating cat saliva in relation to human diseases (Figure 2). Experimental studies investigating the physiology of salivary secretion in a feline model were also included.

## 2 The cat as a model for basic salivary physiology research

The cat is an important model for basic research on salivary secretion. In particular, the influence of the autonomic nervous system on salivary secretion has been investigated in many studies (6–9). The roles of the sympathetic and parasympathetic nervous systems have been studied by severing (10) or stimulation of the corresponding nerves in the model in experimental studies (11). For example, the parasympathetic fibers of the chorda tympani of the facial nerve have been shown to significantly stimulate salivary gland secretion (12). Acetylcholine can be used to experimentally induce changes in electrolyte balance, such as intracellular Na+ and K+ concentrations, in a perfused salivary gland (13–17). The role of hexosamines, calcium and various proteins that may affect secretion were also discussed (18). The endogenous hormone kallikrein, which occurs as a serine protease in the salivary gland, also has a vasodilatory effect and therefore appears to influence salivary secretion (19–22). In addition to basic research on salivary secretion in the feline model, salivary diagnostics appears to be useful for assessing the pharmacokinetics of drugs such as pradofloxacin and doxycycline in the cat (23).

## 3 Infectious diseases of the cat

Infections such as Feline Immunodeficiency virus (FIV) (24–26), Feline leukemia virus (FeLV) (27–29), phlebovirus (30), rabies (31, 32), feline coronavirus (33), feline herpesvirus (26, 34–36), calicivirus (36–38), anellovirus (39, 40), picornavirus (35), feline foamy virus (41), west nile virus (42), hendra virus (43), *bartonella* (44–46), *mycoplasma* (47–51), *helicobacter pylori* (49–51), *staphylococci* (52), *anaplasma* (53), *ehrlichia* (53) and *rickettsia* can be detected by special saliva swab tests (53). Detection can be by polymerase chain reaction (PCR) (30, 46), enzyme-linked immunosorbent assay (ELISA) (54, 55) or immunofluorescence assay (IFA) (56). For diagnostic purposes, rapid saliva test kits, e.g., for antigen detection of FeLV, have also been evaluated (56, 57).

In addition to direct pathogen detection and pathogenesis studies, the prevalence of diseases in specific countries such as *bartonella* in Korea (44), FeLV in New Zealand (54) or in Switzerland (58) and of FIV in Italy has been determined (25). Also of interest was the occurrence of different diseases such as feline foamy virus and FeLV (41) as well as the influence of diseases like FIV on the course of a calicivirus infection (36). Mechanisms of the acute and chronic course of calicivirus infection have been investigated with a view to possible vaccination (38). There are strains of the virus that are resistant to vaccination and may contribute to a chronic course.

## 4 Dental diseases of the cat

Elevated serum and salivary immunoglobulin concentrations were also measured by ELISA in cats with gingivostomatitis (59) or periodontal disease in general (60).

## 5 Further studies on cat saliva

A novel saliva-based test for the diagnosis of food allergy in cats has been studied. It detected antibodies in saliva to certain feed ingredients, including lamb, beef, pork, turkey, fish, wheat, potato, millet, and rice (61).

In a study on blood grouping of cats based on saliva, the authors could not prove any helpful parameters (62).

In a study of blood grouping in cats based on saliva, the authors did not find any useful parameters (63).

## 6 Infections of humans by cat saliva

The diagnosis of infectious agents with zoonotic potential in cat saliva is also of great scientific interest. These include primarily bacteria such as *Pasteurella multocida* (64), which can be transmitted to humans via cat saliva. In addition to the transmission of other pathogens such as *Enterobacteriaceae* (65, 66), *streptococci* and *staphylococci* (64) and *bartonella* (67), the zoonotic potential of the bacterium *Capnocytophaga canimorsus* is discussed, which can cause symptoms such as sepsis and meningitis has been discussed (68, 69).

## 7 Allergies of humans due to cat saliva

Allergens shed by cats that may cause allergic reactions in humans have been investigated in several studies (70, 71). The most important allergen is Fel d1 (72–77). Saliva tests have been performed by ELISA (61, 72, 73, 77), radioimmonoassay (RIA) (76), high performance liquid chromatography (HPLC) (78), immunoelectrophoresis (IEP) (79), a quantitative assay for the detection of monoclonal antibodies, or radioallergosorbent test (RAST), which is also used to detect antibodies to a specific antigen (80, 81). In one study the authors claimed that cat fur was the main source of cat allergens, while another study showed that saliva was the main reservoir (82). The authors of another study considered the skin to be the main source of allergens (74). Thus, the main source of allergens responsible for cat allergy in humans has been controversial for many years. Overall, allergens have been detected in cat excreta such as fur, saliva, skin particles and urine (74, 77, 83, 84).

Recently, new approaches have emerged to investigate Fel d1 blocking antibodies that may reduce cat allergen shedding (75). For example, a clinical trial has already shown that feeding cats a special diet containing these blocking antibodies reduces the rate of antigen excretion (85). From a human health point of view, such a diet therefore appears to be a promising future option for reducing the risk of allergy in the owner.

## 8 Discussion

The aim of this study was to provide an overview through a systematic review of the literature and to identify areas of underuse in cats.

The physiology of salivary secretion in the cat model represents the majority of articles found, 121 out of 225. This is basic research on animal models, with the aim of drawing conclusions about the physiology of other species or humans. These studies can be used in translational medicine, which is why the research interest seems to be particularly high.

With 104 articles, there is still a large number of articles on clinical applications of feline salivary diagnostics. Another major topic was the diagnosis of feline infectious diseases such as FIV and FeLV. This can be explained by the fact that these diseases are very widespread throughout the world (54). The advantage of saliva diagnostics is the high sensitivity for many viruses such as FIV and FeLV, so saliva sampling can be considered a simple alternative to blood sampling in this area of pathogen detection (54).

It should be noted that for some other pathogens, diagnostic results should be interpreted with caution. For example, in a study of *Candidatus Mycoplasma haemominutum* in cats after natural infection, the pathogen was detected in the blood but not in feces and saliva (48). *Candidatus Mycoplasma turicensis* was also detected in cat saliva only in the early phase of infection, not in the late phase (48). The superiority of a saliva test over a blood test depends on the pathogen and must be considered on a case-by-case basis.

In the case of zoonoses transmitted from cats to humans, it should be borne in mind that they are particularly dangerous for young, elderly, pregnant and immunocompromised, e.g., asplenic people. In addition to general hygiene measures such as regular hand washing, vector prophylaxis to prevent communicable diseases should not be neglected. For example, cats should receive regular ectoparasite prophylaxis to minimize the risk of transmission of e.g., *Bartonella henselae* via fleas (86). The transmission of disease from cats to humans through bites is particularly dangerous. These are often underestimated, but can lead to serious consequences and even death (87–89). The most common pathogens include *Pasteurella multocida*, but *Staphylococcus aureus* and a wide range of aerobic and anaerobic pathogens are also commonly found in bite wounds, often requiring appropriate wound revision and antibiotic treatment (89).

Saliva testing can also be useful in feline dentistry, although there are only a few articles on this subject (60, 90). However, it should be noted that infectious diseases such as calici or feline foamy virus can contribute to diseases such as feline gingivostomatitis (91, 92). Fungal diseases can also contribute to gingivostomatitis (93). Although salivary diagnostics are not yet routinely used in veterinary practice, parameters such as oxidative stress have been found to be indicative of tooth resorption and periodontitis (94). Elevated levels of salivary immunoglobulins may indicate feline gingivostomatitis (59). An altered microbiome may also be associated with an increased prevalence of diseases such as gingivostomatitis (95, 96). The pH of saliva and the presence of bacteria such as *Streptococcus mutans* may explain why the prevalence of dental disease such as carious lesions differs between humans and cats. In human medicine, indications of caries can be found by examining the microflora of the saliva (97). Parameters such as alanine asparate aminotransferase may also indicate parodontitis (98). In addition, oral lichen planus disease can be diagnosed based on the microflora and cytokines in the mouth, which has not been directly described in cats (99). Several studies have been conducted to diagnose oral squamous cell carcinoma using saliva (100, 101).

A common disease in small animal medicine is food allergy (63, 87). In a 2018 review article on this topic, the authors concluded that elimination diets are the most important component of diagnosis and treatment (102). However, a year later, a study was published on a novel saliva-based test for the diagnosis of food allergy in cats, which could simplify the diagnosis (61). This test has already been described for dogs. As an elimination diet is often a lengthy and cumbersome procedure, saliva diagnostics could be a faster and less invasive alternative.

It was also possible to find studies in which the test results contradicted the authors' expectations. In a study of AB blood groups, no substances were found in cat saliva that could be used to determine the blood group of the cat (62). Saliva diagnostics is therefore an area of ongoing research.

A comparison of the areas of application for dogs and cats shows that there is still a long way to go in feline medicine. In addition to infectious diseases and allergens (103, 104), stress hormones such as cortisol (105) and vasopressin (106) are measured in dogs. In the present literature search, only one study was found in which salivary cortisol concentrations were measured in cats after administration of alfaxalon or propofol (63). Stress hormones are useful biomarkers of stress and pain, and further studies could be conducted in this area.

Renal values such as salivary urea and creatinine have already been investigated in a pilot study in dogs with chronic kidney disease (107), but no studies have yet been conducted in cats. Inflammatory markers such as C-reactive protein have also been measured in dog saliva (108). In addition, inflammatory parameters such as tumor necrosis factor or various interleukins have already been measured in the saliva of dogs with diabetes mellitus (109). Studies in cats are also lacking in this area. Other known applications in human medicine include genetic analysis (110) and the determination of sex hormones, such as testosterone (111) and progesterone (112) which have not yet been used in feline medicine. There are also studies on the diagnosis of cystic fibrosis (113), Sjögren's syndrome (114), or Prader-Willi syndrome (115) but also tumor diseases [such as breast cancer (116)], pancreatitis (117, 118), diabetes mellitus (119, 120), or sepsis (121–123). Due to the large number of studies in human medicine, only examples can be given here. However, it is clear that the potential of saliva diagnostics in veterinary medicine has not yet been fully exploited.

**Figure 3:**
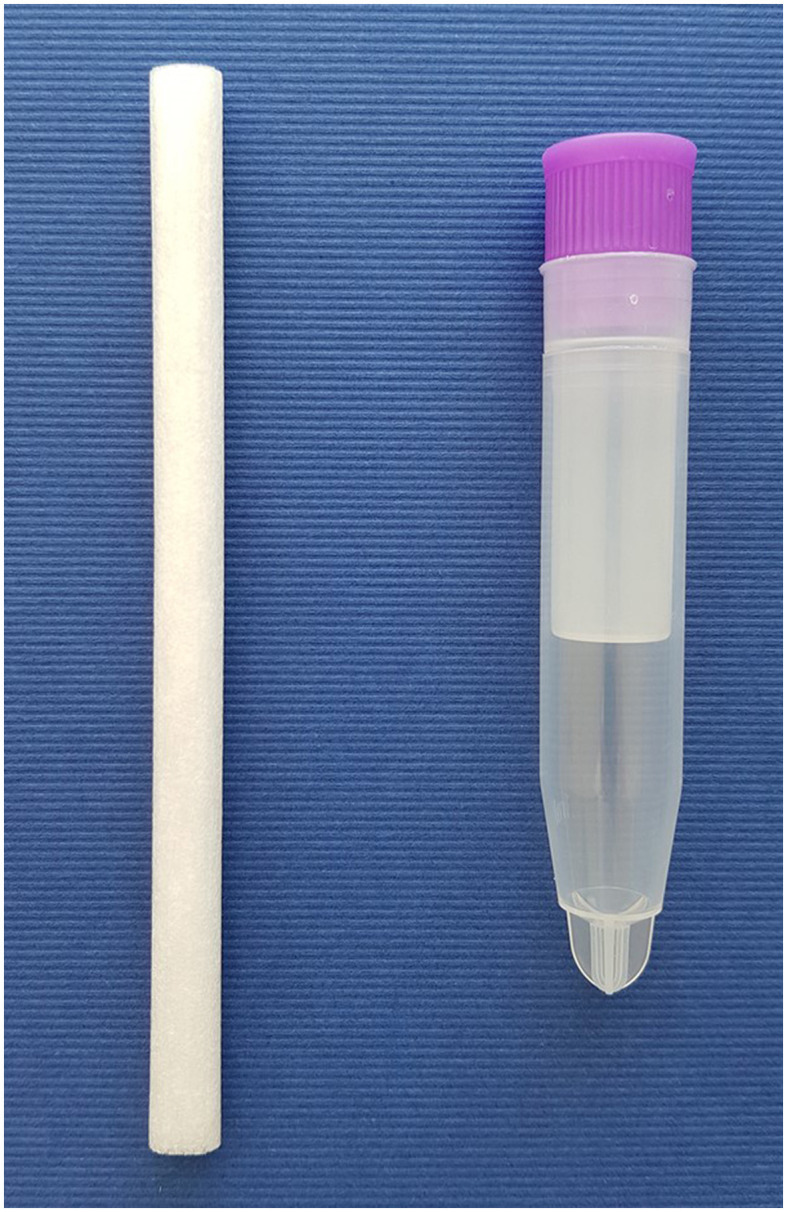
Salivette and corresponding tube for saliva collection.

The advantage of saliva diagnostics is that it may be possible to avoid taking blood from the animal if, for example, the aforementioned infectious diseases are to be detected. In general, salivettes (Figure 1) are often used for saliva diagnostics. In some cases, they need to remain in the animal's mouth for several seconds to collect enough saliva for measurement. However, not only in studies on the detection of infectious agents, but also in studies on stress hormones such as cortisol, a conventional swab was sufficient to collect enough saliva (63). After transferring the salivettes to the appropriate tubes (Figure 3), they are centrifuged and the saliva can then be pipetted and stored until analysis. It should be noted that there is no standardized protocol for salivary diagnostics in cats. The amount of saliva will depend on the parameter being assessed. Saliva samples may be collected in the awake state or under sedation, although certain medications may stimulate salivary secretion. The extent to which stimulation of salivary secretion may affect the test result must be considered for each parameter. The concentrations of the parameter to be determined are usually much lower in saliva than in blood. This is also a disadvantage of saliva diagnostics.

In the present study, the search was carried out using the Pubmed database only. Databases such as Scopus, Web of Science and Google Scholar were not searched, which is a limitation of the work. Nevertheless, a total of 225 articles provided an overview of the relevant areas of application of salivary diagnostics in cats. With 148 articles, a large number of studies had to be excluded. Due to the unspecific search for “cat saliva”, for example, articles on “catfish” and “wild cats” were found, which did not fit the topic. Articles on saliva studies of people with cat allergy and saliva studies of ticks, which can transmit infectious agents to cats, were also excluded (124). Articles were also excluded if they assessed the epidemiology of, for example, pasteurella or rabies in different countries, without examining cat saliva (125). Similarly, articles sialoceles (126), adenocarcinoma of the salivary gland (127) and scintigraphy for thyroid function had to be excluded as well, because saliva was not examined (128). One article on the pancreas described physiological processes that could also be found in other exocrine glands, such as the salivary glands (129), but as salivary diagnostics was not the primary concern, these articles were also excluded.

Due to the large number of search results, the topics could only be summarized and illustrated with examples in this paper. The focus of the studies presented was on frequently investigated areas of application that are of great scientific interest, even though by far not all the possibilities are fully exploited in feline medicine, such as the measurement of stress hormones to assess stress and pain, as is already done in dogs. Overall, salivary testing in cats can be useful in many areas, and in some cases can even save the patient from having to take a blood sample.

## Author contributions

MS: Conceptualization, Data curation, Formal analysis, Investigation, Methodology, Project administration, Software, Writing—original draft. AM-L: Supervision, Writing—review & editing.
